# Modeling tritium release to the atmosphere during the Fukushima Daiichi Nuclear Power Plant accident and application to estimating post-accident water system transit times

**DOI:** 10.1007/s11356-025-35919-1

**Published:** 2025-01-16

**Authors:** Alexandre Cauquoin, Maksym Gusyev, Hayoung Bong, Atsushi Okazaki, Kei Yoshimura

**Affiliations:** 1 Institute of Industrial Science (IIS), The University of Tokyo, 5-1-5 Kashiwanoha, Kashiwa City, 277-8575 Chiba Japan; 2 Institute of Environmental Radioactivity (IER), Fukushima University, 1 Kanayagawa, Fukushima City, 960-1296 Fukushima Japan; 3 NASA Goddard Institute for Space Studies, 2880 Broadway, New York, 10025 NY USA; 4 Institute for Advanced Academic Research/Center for Environmental Remote Sensing, Chiba University, 1-33 Yayoi-cho, Inage, Chiba City, 263-8522 Chiba Japan

**Keywords:** Tritium, Fukushima, Atmosphere general circulation model, Water transit time, MIROC5-iso model

## Abstract

During the Fukushima Daiichi Nuclear Power Plant (FDNPP) accident on March 11, 2011, radionuclides such as tritium were released into the environment across Japan, obscuring the natural background signal of tritium in precipitation. This anthropogenic component was rapidly washed out by precipitation according to measurements in Japan. However, the impact of the accident on the natural tritium-based estimation of water system transit times in Fukushima and other prefectures in Japan remains uncertain. For the first time, anthropogenic tritium from the FDNPP accident together with natural tritium were simulated in an atmospheric general circulation model with a good ability to represent tritium variations in daily and monthly precipitation. For the FDNPP accident, we estimate the maximum tritium atmospheric emission of 0.815 PBq with a tritium in precipitation peak of 68.7 Bq/L (582 tritium units) on March 2011 at Fukushima, which are consistent with previous estimations. Using our modeled outputs with tritium measurements, we improve tritium-tracer application for estimating mean transit times of Fukushima surface and groundwater systems impacted by the anthropogenic tritium from the FDNPP accident. While the anthropogenic impact of the FDNPP accident was limited compared to the tritium peak due to the thermonuclear testing, globally modeled tritium in precipitation is useful to apply for other areas of tritium-tracer studies.

**Supplementary Information:**

The online version contains supplementary material available at 10.1007/s11356-025-35919-1.

## Introduction

The Level 7 accident on the International Nuclear Event Scale at Fukushima Daiichi Nuclear Power Plant (FDNPP, [37.42$^{\circ }$ N; 141.03$^{\circ }$ E]) occurred following the Tōhoku earthquake off Japan’s Pacific coast and the subsequent tsunami on March 11, 2011 (IAEA 2011). During the accident, radioactive nuclides, including $^3$H, were released into the environment (Steinhauser 2014). For example, iodine-131 and cesium-137 were detected 1 month later in 7 and 6 prefectures across Japan (Doi et al. 2013; IAEA 2011), respectively. Natural tritium produced in the atmosphere and anthropogenic tritium released by nuclear facilities (Feng et al. 2023) and accidental releases take the form of tritiated water vapor (HTO) or, to a lesser extent, of tritium gas (T$_2$) or tritiated methane (CH$_3$T) (IAEA 2014). In its HTO form, tritium enters the hydrological cycle and is carried by precipitation into the ocean, rivers, and groundwater. Atmospheric releases of radioactive nuclides by nuclear power station accidents occur near the surface and are then rapidly washed out by precipitation (Povinec et al. 2013; Yoshikane et al. 2016). Following the FDNPP accident, the radionuclides were deposited on the soils and surface water bodies of Fukushima Prefecture, while no tritium measurements in precipitation were taken until radiological safety conditions had been met.

According to previous studies (Povinec et al. 2013; Kaizer et al. 2018), between 0.1 and 1 PBq of tritium has been released into the Pacific Ocean through direct liquid discharges into the sea and atmospheric deposition. Direct tritium runoff, based on the concentration ratio of tritium to cesium-137 in coastal (offshore) monitoring, was estimated at $\sim$0.05 PBq (Takahata et al. 2018). In comparison, the global tritium inventory is estimated at 1275 PBq (UNSCEAR 2000). However, these estimations of tritium released by the Fukushima accident are subject to uncertainties because they are based on a few tritium measurements in North Pacific Ocean and on estimations through tritium/cesium-137 ratios and do not allow to unambiguously distinguish atmospheric inputs and direct liquid discharges.

Tritium radionuclide with a half-life of 12.32 ± 0.02 years (Lucas and Unterweger 2000) is naturally produced by the interaction of galactic cosmic rays (GCRs, which are high-energy charged particles and their secondary products) with nitrogen atoms of the upper atmosphere. Natural tritium concentration in precipitation can vary from 0.5 tritium units (TU; 1 TU corresponds to a $^3$H/H ratio of 10$^{-18}$ and 0.118 Bq/L) in low-latitude regions (Libby 1954) to around 60 TU (or 7.1 Bq/L) in Antarctica (Jouzel et al. 1979, 1982). Large amounts of anthropogenic tritium from thermonuclear bomb tests were injected into the atmosphere, mainly in the stratosphere part during the 1950–1960 s. These tests have caused a “bomb-peak” that led to tritium concentration of around 10,000 TU (or 1180 Bq/L) in Northern Hemisphere precipitation, with a tritium peak of 1680 TU (or 198 Bq/L) measured in monthly precipitation of March 1963 in the Tokyo area (IAEA/WMO 2024). Since the signing of the Partial Nuclear Test Ban Treaty in 1963, the anthropogenic tritium level in precipitation has continuously decreased due to radioactive decay and mixing in the water cycle. After several decades, tritium content in precipitation has decreased to a level approaching that of the pre-bomb period (Terzer-Wassmuth et al. 2022). This return to natural values occurred more rapidly in the Southern Hemisphere and in coastal areas of the Northern Hemisphere (Tadros et al. 2014; Stewart et al. 2017; Gusyev et al. 2019; Feng et al. 2019; Feng and Zhuo 2022).

Measurements of natural tritium content in precipitation and headwater catchments have been used to understand water circulation in wells, freshwater, and geothermal springs (Cartwright and Morgenstern 2018; Gusyev et al. 2016; Stewart et al. 2018; Chatterjee et al. 2019) and to improve calibration of 3-D groundwater flow and tritium transport models (Gusyev et al. 2013, 2014). To estimate water transit times, United States Geological Survey (USGS) TracerLPM program (Jurgens et al. 2012) requires tritium measurements in monthly precipitation, which is the smallest time step, and utilizes long-term continuous data of GNIP Vienna and Ottawa stations (IAEA/WMO 2024). However, the long-term continuous time series of $^3$H in monthly precipitation are generally unavailable in most areas in the globe, and artificial tritium released from nuclear facilities and accidents confound the use of tritium as water transit time tracer for age dating studies in Asia (Gusyev et al. 2019). Therefore, it may be necessary to reconstruct a time series for a specific location using the complete historical data from several periods at the reference site such as the Tokyo area and scale it to the sampling locations (Gusyev et al. 2016; Chatterjee et al. 2019). As a result, it is critically important to quantify anthropogenic tritium released due to the FDNPP accident to utilize natural tritium-tracer for estimating water transit times and groundwater storage volume (Gusyev et al. 2019).

Considering the issues described above, our study aims to answer the following question: what is the impact of the FDNPP accident on water transit times and ground water storage estimations, based on natural tritium content in water in Fukushima and other prefectures in Japan? To address it, we need to assess the amount of tritium injected into the atmosphere during the Fukushima accident and precipitated into the terrestrial water cycle across Japan. In this respect, modeling tritium in a global climate model is one way of solving this problem. In this study, for the first time, we present the HTO modeling during and after the Fukushima accident in an isotope-enabled Atmospheric General Circulation Model (AGCM). First, we briefly describe the model used, the tritium input functions from the Fukushima accident and the observations used for model evaluation. Then, we evaluate the modeled tritium in precipitation time series. Next, we discuss the impact of the Fukushima accident on the estimations of water transit times and storage in terrestrial waters. Finally, we conclude the article with some remarks and perspectives.

**Fig. 1:**
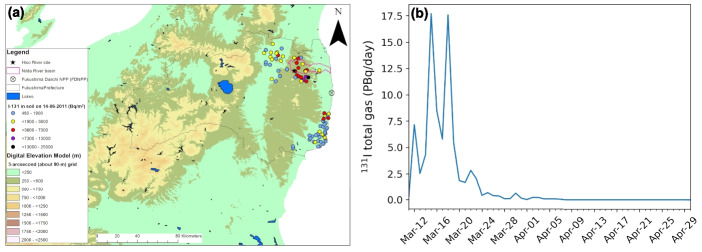
**a** Map of estimated $^{131}$I in soil as of 14 June 2011 (colored circles) around Fukushima area. Hiso River site, Niida River basin, and FDNPP are shown as star, purple contours, and circle cross, respectively. **b** Total gas $^{131}$I emissions during the FDNPP accident from March 11 to April 30, 2011, estimated by Katata et al. (2015)

## Numerical models and simulation setup

### MIROC5-iso atmospheric model

This study uses MIROC5-iso (Okazaki and Yoshimura 2019), which is the atmospheric-land component of the fifth version of Model for Interdisciplinary Research on Climate, Earth System Model (MIROC5) (Watanabe et al. 2010), equipped with stable water isotopes. The implementation of natural tritiated water HTO and the specificities of the model, summarized hereafter, have been described in detail by Cauquoin et al. (2024) and the references therein.

MIROC5 uses the Chikira-Sugiyama cumulus scheme (Chikira and Sugiyama 2010), which is an entraining plume model, for convective parameterization and a semi-Lagrangian scheme for the advection of tracers including the water in its vapor and condensed states (Lin and Rood 1996). Most fractionation in phase transitions is assumed to occur at thermodynamic equilibrium. Kinetic fractionation occurs during surface evaporation from open water, following Merlivat and Jouzel (1979). Evaporation and isotopic exchange from falling droplets into unsaturated air were implemented according to the methods of Stewart (1975) and Yoshimura et al. (2008).

The land-surface component of MIROC5 consists of an independent model called Minimal Advanced Treatments of Surface Interaction and RunOff (MATSIRO) (Nitta et al. 2014; Takata et al. 2003). The kinetic and equilibrium fractionation of water isotopes are considered at any type of surface (i.e., soil, canopy, and snow) during water exchanges with phase changes between the atmosphere and land-surface components such as soil surface evaporation, vegetation transpiration, or snow sublimation. More details can be found in Yoshimura et al. (2006).

The cosmogenic production of natural tritium changes due to modulation of GCRs by solar activity and Earth’s geomagnetic field are considered according to the calculations of Poluianov et al. (2020), along with tritium radioactive decay. We assume that one $^3$H atom produced corresponds to one HTO molecule. The physical and dynamical descriptions of the HTO molecule are the same as for the stable water isotopologues H$_2$ $^{18}$O and H$^2$H$^{16}$O in MIROC5-iso, which makes HTO subject to fractionation processes at each phase change due to the differences of mass and symmetry.

### Tritium inputs estimation from the FDNPP accident

There is no available data or reconstruction regarding the release of tritium in the atmosphere from the FDNPP at the time of the accident. However, Katata et al. (2015) reconstructed hourly iodine-131 ($^{131}$I) releases for the period March 11 to April 30, 2011. For that, they used a reverse estimation method, which calculates the $^{131}$I release rates by comparing $^{131}$I air concentration measurements or its dose rate in the environment (Fig. 1a) with those calculated by atmospheric and oceanic transport, dispersion, and deposition models. Moreover, Maruoka et al. (2017) showed that iodine-129 ($^{129}$I) data correlates with $^3$H data in precipitation and that $^{131}$I and $^{129}$I have a similar distribution and behavior in the environment. Therefore, to simulate the anthropogenic tritium in water vapor and precipitation due to the FDNPP accident, the radionuclide amount and release duration were provided as input to MIROC5-iso atmospheric tritium release time series based on atmospheric total gas emissions of $^{131}$I source term from Katata et al. (2015). This method adopts a one-to-one conversion from released $^3$H gas to released HTO, based on the upper limit of the $^{131}$I release. The different behavior and characteristics of $^{131}$I in the environment in comparison to $^3$H do not impact the modeling of HTO by MIROC5-iso. According to Katata et al. (2015) source term reconstruction, 81.5 PBq of $^{131}$I gas were emitted into the atmosphere during the accident period, which is 2–3 orders of magnitude larger than the emissions of $^3$H during the same period (between 0.05 and 0.95 PBq). In this study, we calculated daily release from the $^{131}$I release reconstruction from Katata et al. (2015), as shown in Fig. 1b, and divided the $^{131}$I release time series with a constant factor between 1000 and 100 (see details in the next section) to match tritium measurements in daily and monthly precipitation, while accounting for their $\pm 3\sigma$ uncertainty. Consequently, the total anthropogenic tritium input forcing into MIROC5-iso is between 0.0815 and 0.815 PBq, which allows for the inclusion of the uncertainty range due to the various reactions and processes simplified in this study. Finally, the ensemble of MIROC5-iso simulations results (Table 1 and results section) fits within the range of uncertainty measurements.

### Simulations scenario of the FDNPP accident

We performed five MIROC5-iso simulations over the period 2011–2021 (Table 1), which are extensions of the CRAC_3H_solar simulation (i.e., with CRAC:3 H natural tritium production rates Poluianov et al. 2020) from Cauquoin et al. (2024). To evaluate the impacts of anthropogenic tritium atmospheric emissions due to the Fukushima accident on the modeled tritium in precipitation, we built four tritium input forcings by dividing the $^{131}$I total gas emissions reconstruction by 100, 200, 500, and 1000. The corresponding simulations are called div100, div200, div500, and div1000, respectively. According to Katata et al. (2015), the release height of $^{131}$I gas was between 20 and a few hundred meters high at maximum. Therefore, each of the anthropogenic tritium forcing for MIROC5-iso was assumed to be uniformly distributed mainly in the first vertical model layer above the surface only (representing the first 60 m) and occasionally in the second layer as well, at the horizontal mesh closest to the FDNPP ([37.68$^{\circ }$ N; 140.625$^{\circ }$ E]). Reconstructed daily values of anthropogenic tritium input forcings, expressed in kg $^3$H, are given in Supporting Information (Dataset S1). One control simulation without anthropogenic tritium forcing, named ctrl, was performed as well.

**Table 1:** Summary of the different MIROC5-iso simulations and their specifications (name, division factor applied on the $^{131}$I time series, and total anthropogenic tritium injected in the atmosphere)

	Division factor applied	Anthropogenic tritium
Simulation name	on $^{131}$I time series	inventory (PBq)
ctrl	−	0
div100	100	0.815
div200	200	0.4075
div500	500	0.163
div1000	1000	0.0815

Except for the additional anthropogenic tritium forcing, all MIROC5-iso simulations have a similar experimental design than the simulations from Cauquoin et al. (2024). They were performed at T42L40 spatial resolution (approximately 2.8$^{\circ }$ horizontal resolution and 40 vertical levels up to 3 hPa). MIROC5-iso simulations were nudged to the U and V components of winds from JRA-55 reanalyses (Harada et al. 2016; Kobayashi et al. 2015) every 6 h for the period of 2011–2021. The 6-hourly mean sea surface temperature (SST) and sea ice area fraction fields from the JRA-55 reanalyses were applied as sea surface boundary conditions. Orbital parameters and greenhouse gases concentrations were set to the values of the corresponding model years following the Coupled Model Intercomparison Project Phase 6 protocol (CMIP6 Eyring et al. 2016). The nudging to reanalyses, in addition to provide model outputs under the same weather conditions than at the sampling time of the observations, allows to reduce model internal variability (Bong et al. 2024). However, the choice of reanalyses for nudging and sea surface forcing impacts the model results. Therefore, to evaluate model uncertainties, we performed the same set of simulations as in Table 1 but by using the ERA5 reanalyses (Hersbach et al. 2020) instead of JRA-55 for nudging, SST and sea ice area fraction boundary conditions (see Figs. S1 and S2 in Supplementary Information). For tritium sea surface boundary concentrations, we assumed constant values over time using the map reconstructed by Cauquoin et al. (2016) from available tritium data for the year 2004, which is the most recent year available in this reconstruction. The tritium concentration in surface seawater off the coast of Fukushima in this reconstruction is around 0.7 TU, which is comparable with values ranging from 0.5 to 1.3 TU measured in seawater samples collected just after the accident offshore Fukushima (Povinec et al. 2013). Moreover, Cauquoin et al. (2024) showed that FDNPP accident has a low impact on tritium concentration in surface seawater using a reconstructed discharge tritium inventory (Machida et al. 2022) as an input to the ocean general circulation model COCO4.9 (CCSR Ocean Component Model version 4.9, see Hasumi 2006). Therefore, we do not expect large biases in our modeled results considering this rather low increase in tritium concentration of surface ocean water around the eastern coast of Japan. Should the actual tritium concentration in nearby surface seawater were to be slightly higher than that prescribed in our experimental design, the modeled decline in tritium in precipitation over the 3-month period following the FDNPP accident may be slightly overestimated.

**Fig. 2:**
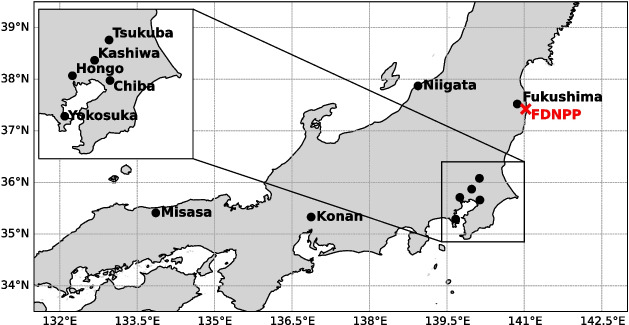
Locations of tritium sampling sites in daily and monthly precipitation (black dots) and FDNPP site (red cross)

### Lumped-parameter model

Tritium-tracer transit times were estimated by simulating the time-dependent tritium concentration $C_{out}(t)$ [TU] at the time of sampling *t*, at the groundwater discharge point such as a well, spring, or river with the convolution integral in the lumped-parameter models (LPMs) in TracerLPM (Małoszewski and Zuber 1982; Jurgens et al. 2012):

1 $$\begin{aligned} C_{out}(t) = \int _{-\infty }^{t} C_{in}(\tau ) e^{\lambda (t-\tau )} g(t-\tau )d\tau \ \end{aligned}$$

where $C_{in}(\tau )$ [TU] is the tritium-tracer concentration in the water that entered the system at the date $\tau$, $\lambda$ [year$^{-1}$] is the tritium decay constant of 0.056262 [year$^{-1}$] estimated from the tritium half-life of 12.32 years, and $g(t-\tau )$ [-] is the weighting (or system response) function that has a pre-defined distribution of transit time such as the Exponential Mixing Model (EMM) or the Exponential-Piston Model (EPM). In the TracerLPM model, tritium input requires the continuous time series available at the nearby GNIP station with the long-term tritium record in monthly precipitation, which is scaled using the short-term tritium measurements and bias-corrected by local precipitation (Chatterjee et al. 2019; Gusyev et al. 2019). For the Fukushima area, tritium time series in precipitation was constructed in this study by combining three monthly records: (1) the continuous time series of measured tritium in monthly precipitation of the Tokyo area up to December 2010 (Gusyev et al. 2016; 2) the modeled tritium time series from January 2011 to October 2012 with and without the FDNPP peak presented in this study; and (3) the measured tritium at Namie town, Fukushima Prefecture, from October 2012 to January 2016 (Yamada et al. 2024). Using the statistically significant linear regressions, the scaling factor is calculated based on the latitude leading to the latitudinal scaling of 1.33 from the Tokyo area (35.5$^{\circ }$ N) to Fukushima area (37.5$^{\circ }$ N) (see Fig. 5d in Gusyev et al. 2019). The large range of the latitudinal scaling uncertainty, which is between 0.38 and 2.38, is constrained by the tritium measurements in local precipitation for the Fukushima area reported by Yamada et al. (2024). Following Gusyev et al. (2019), the precipitation-weighted tritium concentrations measured in monthly precipitation at Namie town, Fukushima Prefecture, from October 2012 to December 2016 were compared to the tritium levels observed in the Tokyo area for the same period. This results in a latitudinal scaling of 1.31, which was used in this study. This value is similar to the scaling factor of 1.33 estimated by Gusyev et al. (2019).

**Table 2:** Specification of selected tritium data in river, spring, and artesian well water in the Niida River basin of the Minamisoma area (type, date, coordinates, $^3$H measurement ± $\sigma$ uncertainty, and reference)

Sampling site	Type	Date	Lat. ($^{\circ }$ N)	Lon. ($^{\circ }$ E)	$^\textbf{3}$H conc. (TU)	Ref.
Hiso River monitoring site	low river spring	2011-08-10	37.65	140.70	9.57 ± 0.17	1
		2013-08-29	37.65	140.70	4.41 ± 0.17	1
Minamisoma area spring	groundwater spring	2013-05-06	37.63	141.01	11.36 ± 0.76	2
		2014-04-10	37.63	141.01	8.89 ± 0.76	2
Minamisoma artesian well	groundwater	2013-04-19	37.60	141.02	3.73 ± 0.84	2
		2014-04-10	37.60	141.02	3.39 ± 0.68	2

References 1 and 2 correspond to Ueda et al. (2015) and Yabusaki et al. (2015), respectively

Since the LPMs represent the pre-defined distribution of the transit times and is used to indicate the young water fraction, which is an important indication of groundwater quality (Stewart et al. 2017), these distributions are evaluated using the probability density and cumulative frequency distributions in the TracerLPM (Jurgens et al. 2012). In the LPM, the system response function of the EMM that represents recharge for the entire drainage area of the groundwater discharge point is defined as

2 $$\begin{aligned} g(t-\tau ) = \frac{1}{\tau } \ exp\left( -\frac{(t-\tau )}{T} \ \right) \end{aligned}$$

where *T* [year] is the water mean transit time (MTT) that is the only calibration parameter to match simulated and measured tritium values. In the partially confined aquifer, the confined portion that does not receive infiltration is represented by the piston flow model (PFM), which is an instantaneous pulse moving through the system, while the unconfined part of the aquifer is described by the EMM, resulting in the EPM:

3a $$\begin{aligned} g(t\!-\!\tau )&\!=\! \frac{n}{T} \ exp\left( -\frac{n(t-\tau )}{T} \ \!+\! n \!-\! 1 \right) \textrm{for}\ t \!\ge \! T(1\!-\!1/n) \end{aligned}$$

3b $$\begin{aligned} g(t-\tau )&= 0\ \textrm{for}\ t < T(1-1/n) \end{aligned}$$

where *n* [-] is the ratio of the total area (*x*) to the exponential component ($x^{*}$) of the aquifer $n = (x^{*}+x)/x = \mathrm {EPM\ ratio} + 1$ (Jurgens et al. 2012). This EPM ratio is the calibration parameter representing the ratio of the piston flow to exponential components. It is equal to 0 for the EMM and is close to 1 for the nearly piston flow. To evaluate the LPM parameter uncertainty, we utilized the VBA script developed in the TracerLPM (Gusyev et al. 2016) to iteratively evaluate simulated tritium due to the varied MTTs and EPM ratio values. In this study, we varied the EPM ratio from 19 to 0.001, and each of 21 EPM ratio values was iteratively run with MTT from 1 to 201 years with time steps of 0.1 and 1 years. The simulated tritium values were stored in the designated spreadsheet, and the difference between simulated and measured tritium was computed. The combination of MTT and EPM ratio was recorded when the difference was within $\pm 1 \sigma$ of tritium analysis error, which was considered as a good fit. Since the aim of this study was to demonstrate the anthropogenic tritium influence on MTTs, we reported the MTT range for the selected EPM ratio without the full range of MTTs (Chatterjee et al. 2019). The estimated MTT (year) can be used with discharge *Q* (m$^3$/s) measurement to find the groundwater storage, *V* [m$^3$], as $V = \textrm{MTT} \times Q \times 86400 \times 365$ and the estimated groundwater storage provides the initial conditions to evaluate changes in groundwater storage in different climates (Gusyev et al. 2016).

### Tritium measurements for model evaluation

#### Daily and monthly precipitation

To evaluate our model results and determine the amount of anthropogenic tritium injected into the atmosphere during the FDNPP accident, we compiled tritium concentrations measured in daily and monthly precipitation samples in Japan (Fig. 2). For daily values, we selected tritium measurements in individual rainfall events from Matsumoto et al. (2013), sampled from March to May 2011, in order to evaluate daily mean variations of tritium in precipitation modeled by MIROC5-iso. The stations closest to the FDNPP are located in Hongo (Tokyo), Kashiwa (Chiba Prefecture), Yokosuka and Tsukuba (Ibaraki Prefecture) at the southeastern tip of Japan (see zoomed-in map in Fig. 2). The furthest stations are located to the south-east of the FDNPP, in Konan and Misasa (Matsumoto et al. 2013). Moreover, we used observations of tritium in monthly precipitation sampled from year 2011 to early 2016 in Niigata city, Niigata Prefecture (Wang et al. 2016), and Chiba, Chiba Prefecture (Japan Chemical Analysis Center (JCAC) 2024), at the west and south-west of the FDNPP, respectively. We also used monthly tritium in precipitation data from Namie, Fukushima Prefecture (Yamada et al. 2024), which carried out the sampling from the end of 2012. As a result, the tritium peak due to the accident could not be measured at this location requiring numerical modeling. In addition, the strong tritium peak and subsequent decrease measured at Tsukuba (Matsumoto et al. 2013), Niigata, and Chiba allowed us to test the moisture transport dynamics of MIROC5-iso. The weaker response of tritium in precipitation in sites like Konan and Misasa are useful to evaluate the horizontal spread of the anthropogenic tritium signal in MIROC5-iso.

#### River, spring, and artesian well

To constrain MTT estimations with TacerLPM model (equation 1), we chose the Niida River basin (purple contours in Fig. 1a), which is located within 30 km of the FDNPP, with tritium river water measurements from 2011 to 2014 by Ueda et al. (2015) and tritium measurements in groundwater of springs and wells in 2013 and 2014 by Yabusaki et al. (2015). The summary of three sites and selected tritium measurements is reported in Table 2. From Ueda et al. (2015), we selected two tritium measurements during low river flows at Hiso River monitoring site, which has a 4.6 km$^2$ catchment area (see black star in Fig. 1a), of 9.57 (± 0.17) TU on 10 August 2011 and 4.41 (± 0.17) TU on 29 August 2013. The decline of the anthropogenic tritium from the FDNPP accident recorded in those two measurements allows to evaluate TracerLPM simulated tritium with and without the FDNPP accident input. For the groundwater pathway, we selected the Minamisoma area spring Table 2, which is located near the Niida River outlet, with tritium measurement of 11.36 (± 0.76) TU on 6 May 2013 and of 8.89 (± 0.76) TU on 16 April 2014 affected by the anthropogenic tritium from the FDNPP accident. In the same Minamisoma area, background tritium measurements of 3.73 (± 0.84) TU and 3.39 (± 0.68) TU were observed in the artesian well on 19 April 2013 and 10 April 2014, respectively.

**Fig. 3:**
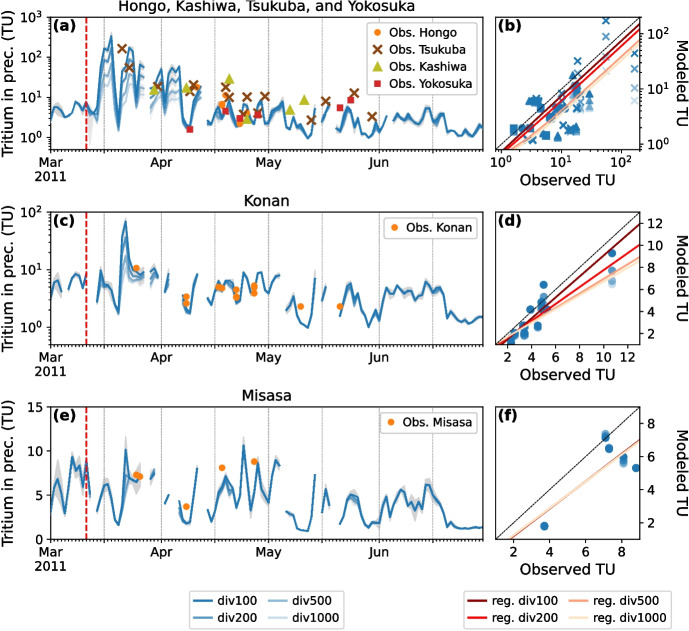
Tritium in precipitation from January to June 2011 in Hongo, Kashiwa, Tsukuba, and Yokosuka (observed and modeled time series in **a**, observed vs. modeled tritium in precipitation values in **b**), Konan (**c** and **d**), and Misasa (**e** and **f**). In time series plots (**a**, **c**, and **e**), blue lines are modeled daily mean values at the nearest grid cell of the observations’ location. Dark to light blue colors indicate the anthropogenic tritium forcing function applied, from a scaling by 100 (div100) to 1000 (div1000). The missing modeled tritium values on certain days are due to the absence of modeled precipitation on those days. Uncertainty ranges of modeled values are represented by shaded gray areas. Colored markers are observed tritium values in individual precipitation events. Vertical red dashed line indicates the day of the FDNPP accident. The closest MIROC5-iso grid cell for the Hongo, Kashiwa, Tsukuba, and Yokosuka sites is the same. In model-data scatter plots (**b**, **d**, and **f**), dark to light blue markers indicate the anthropogenic tritium forcing function applied in the same way as in time series plots (**a**, **c**, and **e**). Model-data linear regression fits with modeled results from div100 to div1000 simulations are drawn by lines ranging from dark to light red, respectively. Linear fits in plot (**b**) were calculated with the decimal logarithm of observed and modeled values (i.e., $\log _{10}(\mathrm {model.\ TU}) = a \times \log _{10}(\mathrm {obs.\ TU}) + b)$

## Results: tritium in precipitation during and after the FDNPP accident

### Tritium concentration in precipitation events

The daily mean tritium in precipitation time series from the MIROC5-iso simulations are evaluated with tritium measurements in precipitation events in Fig. 3. Model-data regression statistics are provided in Supporting Information (Table S1). For Kashiwa and Yokosuka stations considered alone, as well as for Misasa, model-data correlations are not significant ($p > 0.05$). For all other cases, we found better model-data agreement with the div100 simulation, i.e., for the highest tritium emission case (dark red regression line in graphs b and d of Fig. 3, Table S1). The declining trend in anthropogenic tritium observed at Tsukuba (brown crosses in Fig. 3a), from 164.2 (± 8.0) TU on March 21$^{\text {st}}$ to 12.8 (± 0.3) TU on May 15$^{\text {th}}$, is attributed to mixing with surrounding tritium-free oceanic water vapor and precipitation washout. The div100 simulation accurately reproduces this trend (linear regression gradient $a = 0.94$ with 1.0 being the perfect fit, and Pearson correlation coefficient $r = 0.73$), despite the coarse spatial resolution of MIROC5-iso. The conclusions are the same when considering all sites in the southeastern tip of Japan, i.e., Hongo, Tsukuba, Kashiwa, and Yokosuka ($a = 0.75$ and $r = 0.66$ for div100 simulation) (Fig. 3a and b). Our results are significant considering the modeled value uncertainties (shaded grey areas in Fig. 3a), which range from less than 0.2 TU to 76 TU for the background level and the highest value, respectively. Modeled tritium in precipitation variations at the more distant Konan station (Fig. 3c and d) are in good agreement with the observations, too ($a = 0.95$ and $r = 0.93$ for div100 simulation). Although no significant model-data correlations are found for the Kashiwa, Yokosuka (green triangles and red squares in Fig. 3a), and Misasa (Fig. 3e) sites, the mean modeled tritium values are consistent with the observations. Finally, the modeled values in all simulations are very similar with one another from mid-April 2011 onwards, due to the near-total elimination of anthropogenic tritium in precipitation and the application of JRA-55 horizontal winds for nudging.

**Fig. 4:**
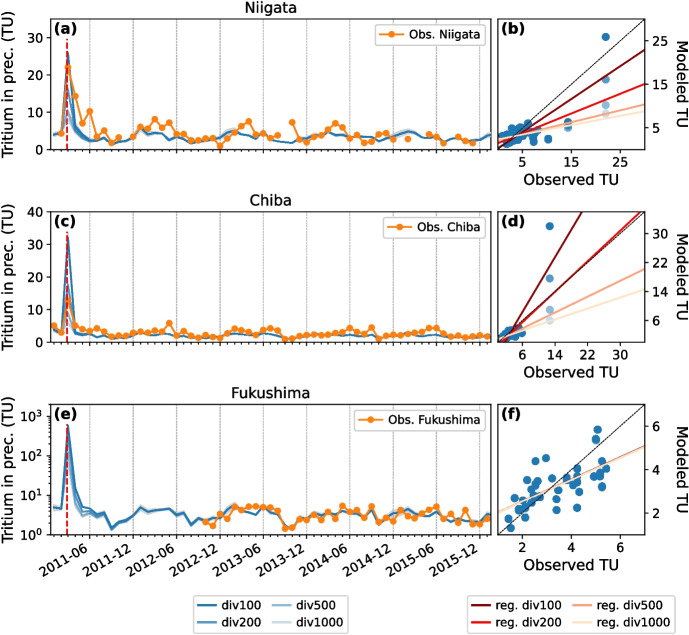
Same as Fig. 3 but for tritium in monthly precipitation from January 2011 to January 2016 in Niigata (observed and modeled time series in **a**, observed vs. modeled tritium in precipitation values in **b**), Chiba (**c** and **d**), and Fukushima (**e** and **f**)

### Tritium concentration in monthly precipitation

The modeled and measured tritium in monthly precipitation time series at Niigata, Chiba, and Fukushima are evaluated in Fig. 4. Model-data regression statistics are provided in Table S2. As for daily tritium time series, best model-data agreement at Niigata site is found for anthropogenic tritium emissions of 0.815 PBq (red dark line in Fig. 4b). Such a forcing leads to an overestimation of March 2011 tritium peak in Chiba (32 ± 2 TU modeled vs. 12.7 (± 0.2) TU observed, see Fig. 4c). As a result, the div200 simulation is in better agreement with the Chiba’s tritium observations (Fig. 4d, $a = 1.05$, $r = 0.85$, peak of 17.6 ± 1 TU). The better agreement with tritium observations at Chiba for anthropogenic tritium emissions of 0.41 PBq instead of 0.815 PBq as at other nearby stations (Hongo, Kashiwa, and Tsukuba, Fig. 3) could be explained by the relatively coarse spatial resolution of our model. For Fukushima Prefecture, the March 2011 tritium peak of 582 ± 9.3 TU is modeled in div100 simulation (Fig. 4e). This simulated tritium peak in monthly precipitation is comparable to the estimated mean tritium concentration of 433 TU, which was reported by Kashiwaya et al. (2017) for the weeks after the FDNPP accident. This estimate was based on groundwater samples collected in 2012 at 25-km south from the FDNPP site, with the highest tritium measurement recorded at 13 TU (Kashiwaya et al. 2017). Similarly, groundwater wells and springs were sampled at the 25-km north of the FDNPP site in 2013 with the highest tritium measurement determined to be 11.36 ± 0.76 TU (Yabusaki et al. 2015; Yabusaki and Asai 2023). For the same div100 simulation, maximum daily mean tritium concentration in precipitation is 3643 ± 621 TU, which is of the same order of magnitude as the highest tritium value of 1559 TU (or 186 Bq/L) measured in a paddle water sample near the FDNPP site by Querfeld et al. (2019). Kashiwaya et al. (2017) estimated the highest tritium concentration in daily precipitation at 1342 TU at the 25 km point on 21 March. Tritium peak value is around twice as low in div200 simulation, which makes it in good agreement with the observations and estimations mentioned above, too. Conversely, we conclude that the peak estimation by Matsumoto et al. (2013) of 130,000 TU at the FDNPP during the accident, which is one order of magnitude higher than the bomb peak, is a significant overestimation. We found similar reasonably good model-data agreements with all MIROC5-iso simulations (*a* is between 0.53 and 0.54, and *r* between 0.65 and 0.66). The reason for this analogous agreement is that measurements at Fukushima are limited to the background level period only, i.e., without the tritium peak due to the FDNPP accident.

**Fig. 5:**
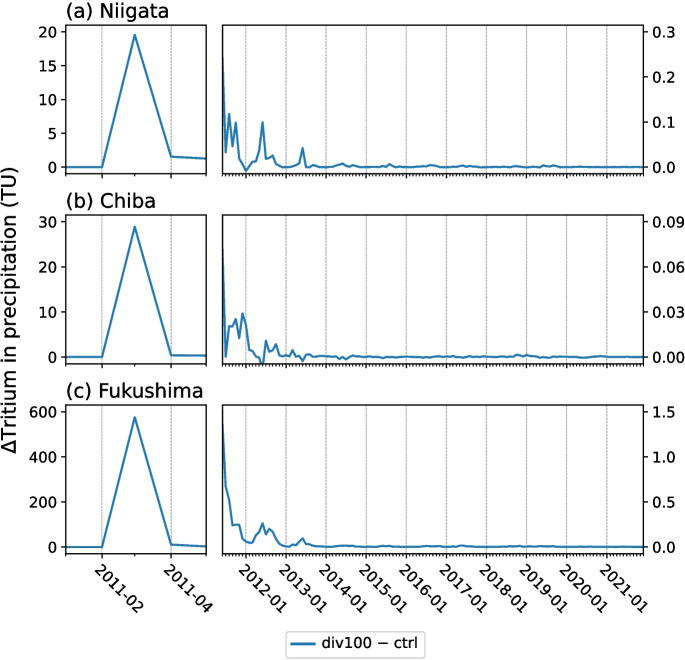
Difference in modeled tritium in monthly precipitation between div100 and ctrl simulations for the sites of **a** Niigata, **b** Chiba, and **c** Fukushima

## Discussion: what is the impact of the FDNPP accident on tritium-tracer application?

Based on our model results, we estimate that between 0.41 and 0.815 PBq of tritium were released into the atmosphere during the FDNPP accident, consistent with the estimate of 0.7 ± 0.3 PBq for the western North Pacific Ocean by Kaizer et al. (2018). Due to predominant westerlies around Japanese coasts, anthropogenic tritium was rapidly washed out from the atmosphere by precipitation in the northwest Pacific Ocean. As a result, modeled tritium in precipitation decreases within 1 or 2 months after the March 2011 peak in all sites. These findings are in agreement with tritium observations by Matsumoto et al. (2013). From June 2011, the impact of anthropogenic tritium emission from FDNPP accident on modeled tritium content in precipitation is already very low, with div100 − ctrl anomalies of up to 0.4, 0.08, and 1.4 TU at Niigata, Chiba, and Fukushima, respectively (Fig. 5). As of 2014, the FDNPP anthropogenic contribution to tritium level can be considered totally insignificant in Japan and elsewhere. Furthermore, the rapid removal of tritium makes the FDNPP anthropogenic forcing a local rather than a country-wide or regional effect, as shown by the lower peak at stations further away from the FDNPP site such as Niigata (Fig. 4a), Konan (Fig. 3c), and Misasa (Fig. 3e). As a result, the FDNPP anthropogenic contribution has no impact on the Asian region for tritium-tracer studies.

**Fig. 6:**
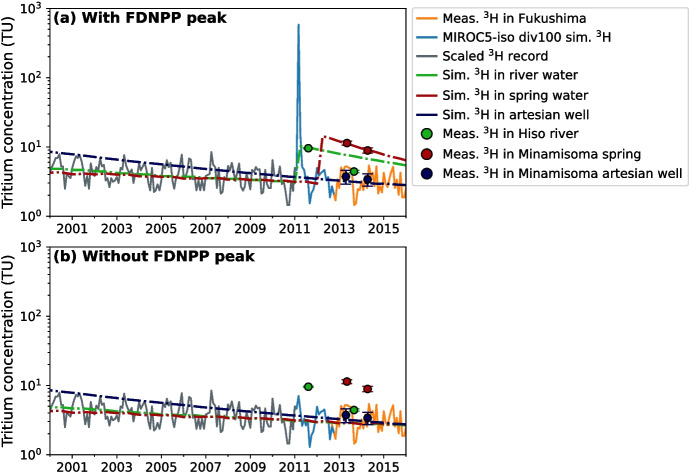
Tritium in precipitation time series used as input $C_{in}(t)$ to TracerLPM model, simulated tritium concentration time series $C_{out}(t)$ at the Fukushima groundwater discharge point and comparison with tritium observations from Niida River basin for the period 2000–2016. Zooms in on the 2010–2016 period are also presented. Grey, blue, and orange plain curves are Tokyo tritium data scaled to Fukushima area, modeled tritium values at Fukushima (div100 in **a**, ctrl in **b**) and measured tritium data from Yamada et al. (2024), respectively, used as $C_{in}(t)$. Dashed green, dark red, and dark blue curves are time series of tritium concentration in river, spring, and artesian well waters, respectively, modeled by TracerLPM ($C_{out}(t)$). Green, dark red, and dark blue circles are measured tritium values in Hiso River, Minamisoma spring, and artesian groundwater, respectively (Table 2). Reconstructions with and without the FDNPP peak are shown in plot **a** and **b**, respectively

Having simulated FDNPP tritium release, we are able to apply both anthropogenic and natural tritium in precipitation for estimating transit times in the Fukushima area (Fig. 6). In panels (a) and (b), the solid blue line corresponds to the MIROC5-iso simulated tritium with (div100 simulation) and without (ctrl simulation) the FDNPP release from Jan. 2011 to Sep. 2012, respectively. The measured tritium in monthly precipitation at Namie town is shown by the solid orange line from Oct. 2012 to Jan. 2016, which is the same data as in Fig. 4e. The grey line displays the tritium in monthly precipitation scaled from the Tokyo area to Fukushima, which is also shown in Fig. S3. Combining simulated, measured, and scaled tritium time series, we obtain the continuous tritium time series in precipitation of the Fukushima area that is used as the input tritium concentration $C_{in}(t)$ in the TracerLPM model. For the Tokyo area measurements, applying this scaling factor increases the tritium monthly precipitation peaks of 1680 TU on March 1963 and 1605 (± 32) TU on March 1964 (IAEA/WMO 2024) to the Fukushima area tritium of 2201 TU and 2103 TU, respectively. As a result, the anthropogenic tritium peak in Fukushima monthly precipitation due to the FDNPP accident in March 2011 is around 3 times smaller than the peak from atmospheric thermonuclear testing in March 1963 (Fig. S3). Therefore, the tritium peak of the FNPP accident has a minor influence across Japan compared with the 1963–64 tritium bomb peak, which still interferes with tritium-transit time interpretation of natural background tritium measurements in river water of Hokkaido, Japan (Gusyev et al. 2016).

The results of the TracerLPM model with and without simulated anthropogenic tritium in precipitation are demonstrated in Fig. 6a and b, respectively. With the exception of the MIROC5-iso simulated tritium in $C_{in}(t)$ (solid blue lines in Fig. 6), the parameters used for the MTT curve fitting in TracerLPM are identical in Fig. 6a and b. Two tritium measurements of the 30-m deep artesian well are illustrated by dark blue circles, and the simulated tritium using the EPM (MTT = 18 years and the EPM ratio = 0.25) is shown by dark blue dashed lines. Since these tritium measurements are considered to be background tritium levels, we find a similar range of estimated MTTs. Using the EPM with a EPM ratio equal to 0.25, the MTT range with the tritium release is between 10 and 98 years with an average MTT of 56.5 ± 20.4 years. Without the tritium release, the MTT range is from 17 to 100 years with an average MTT of 63.5 ± 27.3 years. As detailed in lumped-arameter model section, the tritium measurement uncertainty needs to be small enough for reducing the MTT range estimated from current tritium-tracer studies. By employing low-level enrichment, Gusyev et al. (2024) achieved a one-standard deviation uncertainty of less than 0.2 TU for tritium measurements in river water samples collected on October 12, 2023 near Fukushima city. For such recent tritium measurements in terrestrial waters, the FDNPP accident release of tritium does not affect MTTs estimation in Fukushima and elsewhere, making natural cosmogenic tritium radionuclide a useful water cycle tracer.

Despite the rapid return of tritium concentrations in precipitation to natural background levels after the FDNPP accident, elevated tritium measurements were reported in some springs of the Minamisoma area (dark red circles in Fig. 6) and river water (green circles in Fig. 6) showed between 2011 and 2015 (Table 2). These tritium values were likely affected by the anthropogenic tritium from the FDNPP accident requiring the tritium peak representation to estimate MTTs as shown in Fig. 6b (green and dark red dashed lines). In this case, our simulated anthropogenic tritium in precipitation can be used for estimating MTTs from both anthropogenic and natural tritium, as illustrated by the good fit of simulated and measured tritium in springs and river water (Fig. 6a). A similar good model-data agreement is found if outputs from div100 simulation nudged to ERA5 instead of JRA-55 reanalyses are used in $C_{in}(t)$ (Fig. S4), showing the low impact of MIROC5-iso model uncertainties on the MTTs estimation.

For the elevated tritium contents in spring, we simulate tritium concentrations using the EPM with MTT and EPM ratio equal to 5 years and 0.25, respectively (dashed red lines in Fig. 6). Using an EPM ratio equal to 0.25, we obtain a narrow MTT range between 4.7 and 5.1 years with an average MTT of 4.85 ± 0.13 years. To match elevated tritium content observed in the Hiso River water, we simulate tritium using the EMM with the MTT value of 6.7 years (dashed green line in Fig. 6). The MTT is the sole calibration parameter for the EMM (see Eq. 2), resulting in a MTT range of 6.6 to 6.8 years. Using the spring discharge of 0.004 m$^3$/s with MTT = 4.85 ± 0.13 years, we estimate a groundwater storage of 0.61 ± 0.02 million m$^3$. Using ± 0.1 years MTT uncertainty with the river discharge of 0.12 m$^3$/s, we estimate a groundwater storage of 25.35 ± 0.38 million m$^3$ for the upstream catchment of the Hiso River site. The estimated groundwater storage of the Minamisoma spring is rather small compared with the groundwater storage of the Hiso River catchment, which is similar to the estimated groundwater storage in headwater catchments of Hokkaido, Japan (Gusyev et al. 2016).

In the TracerLPM, the uncertainty of the MTT estimation arises from (1) the parameterization of the selected LPM with two calibration parameters and (2) the tritium input function developed for the study area. For (1), the MTT uncertainty associated with the LPM parameters provides a non-unique fit to tritium measurements at the entire range of LPM parameter values. This is due to the large uncertainties associated with tritium observations. For example, the tritium measurement uncertainty for the Minamisoma spring is ± 0.76 TU, while the uncertainty for artesian well is ± 0.84 and ± 0.68 TU for the years 2013 and 2014, respectively (Table 2). In the case of the Hiso River, the lower uncertainty of ± 0.17 TU results in a reduction of the number of fitted MTTs. Moreover, a significantly reduced uncertainty of one order of magnitude due to the ultra-low tritium measurement results in a single MTT solution in the Hokkaido River catchments, Japan, while non-unique fits of MTTs are due to the bomb-peak tritium that still contributing to the MTT uncertainty (Gusyev et al. 2016). For (2), the source of uncertainty may arise due to the statistical transfer of the continuous tritium time series from a known location to the study area, which lacks tritium measurements in local precipitation. Regarding Japan, the latitudinal scaling approach is the most robust method given the climate similarities across the country and available short-term tritium measurements in local precipitation for the scaling adjustments (Gusyev et al. 2016). In this study, Gusyev et al. (2019) calculated a scaling factor of 1.31 from the Tokyo area to Fukushima and the additional incorporation of tritium measurements in monthly precipitation at Namie town, Fukushima prefecture (Yamada et al. 2024), results in an updated scaling factor value of 1.33 for the tritium time series of the Tokyo area from 1950 to 2010. In addition, we utilize tritium in precipitation to illustrate the impact of FDNPP accident as a case of study of a combined anthropogenic and natural tritium usage for the MTT estimation. This assumption leads to less than 20% uncertainty due to infiltrated water in the northern Japan. For example, Gusyev et al. (2016) derived a scaling factor of 2.1 for infiltrated water using tritium measurements in wine samples from Hokkaido, while the scaling factor of 2.4 was estimated for tritium monthly precipitation of Hokkaido area (Gusyev et al. 2019). For semi-arid and arid areas, it is also important to estimate infiltrated water tritium that is used as the tritium input function in TracerLPM (Chatterjee et al. 2019). When the study area is located in a different climate than the site with long-term continuous tritium time series, the scaling factor necessitates the application of a monthly precipitation weighting to the annual tritium values (Chatterjee et al. 2019).

## Concluding remarks and perspectives

This study is the first attempt to simulate the anthropogenic tritium from the FDNPP accident together with natural tritium by an AGCM. Despite the coarse spatial resolution of MIROC5-iso, we found relatively good agreement with tritium observations in daily and monthly precipitation in Japan for anthropogenic tritium atmospheric emissions between 0.41 and 0.815 PBq. The March 2011 tritium peak at Fukushima modeled by MIROC5-iso is comparable to previous estimations based on groundwater and spring samples. The use of ERA5 reanalyses instead of JRA-55 ones for the nudging does not change our conclusions (Figs. S1 and S2), and we found a slightly better agreement with tritium observations when using JRA-55 reanalyses for nudging, especially for the sites of Tsukuba, Hongo, Konan sites, and the Hongo + Kashiwa + Tsukuba + Yokosuka combination (see Tables S1 and S3). Since this study quantified the anthropogenic tritium release due to the FDNPP accident, it was possible to use MIROC5-iso outputs together with tritium measurements in Fukushima coastal area precipitation for constructing long-term tritium in monthly precipitation time series. This constructed tritium time series was used as an input in the LPM TracerLPM, with the objective to improve the MTT estimations for the post-accident surface and groundwater tritium measurements that were affected by the FDNPP tritium release. The post-accident tritium measurements not affected by the anthropogenic tritium release during the FDNPP accident have no impact on the model-data fitting parameterization of TracerLPM. Consequently, there is no major effect on the MTT estimations in the recent tritium measurements in Fukushima (Gusyev et al. 2024). Therefore, the combined use of this study model outputs with tritium measurements in local precipitation provides the required long-term continuous time series of tritium in monthly precipitation for future tritium-tracer studies in Fukushima and other areas.

This study focused on estimating $^3$H release following the FDNPP accident in the atmosphere, with a particular emphasis on terrestrial water cycle impacts. Thanks to estimated ocean discharge inventory of tritium from the FDNPP site (Machida et al. 2022), it is now possible to model and evaluate the impact of the FDNPP accident on $^3$H concentrations in the Pacific Ocean (Cauquoin et al. 2024), too. The use of an atmosphere-ocean coupled model would make it possible to model tritium movement in both water vapor and ocean water as well as the dynamics of exchanges within and between the atmosphere, land, and ocean. Such coupling would permit the evaluation of the impacts of $^3$H concentration changes in precipitation and runoff following the FDNPP accident on $^3$H seawater concentration in the northwest Pacific Ocean, in addition to the available tritium observations. Moreover, it would dispense the need to prescribe uncertain tritium boundary conditions at the sea surface, as in our current model configuration. Finally, although the results of the MIROC5-iso model agree well with the tritium observations during the FDNPP accident, the necessity of a higher spatial grid resolution would improve tritium modeling capability for the MIROC5-iso model. In this regard, the future coupled simulations of tritium will be performed at higher spatial resolution.

## Supplementary Information

The following files are in supplementary material:

- SI_tritium_fukushima_accident.pdf: model-data com-parison for tritium concentration in daily and monthly precipitation using the MIROC5-iso simulations nudged to ERA5 (Figs. S1 and S2); simulated tritium concentrations at the Fukushima groundwater discharge point for the period 1950–2016, and by using results from div100 simulation nudged to ERA5 (Figs. S3 and S4); and model-data correlation statistics for all the simulations (Tables S1 to S4).
- Dataset S1: Table of anthropogenic tritium daily re-lease (CSV file), based on reconstructed iodine-131 total gas emissions from Katata et al. (2015), used as inputs for MIROC5-iso. It is also available in the Zeno-do database (https://doi.org/10.5281/zenodo.14020821, Cauquoin et al. 2024).

## Supplementary Information

Below is the link to the electronic supplementary material.

Supplementary file 1 (pdf 506 KB)

Supplementary file 2 (csv 3 KB)

## Data Availability

The GNIP data (IAEA/WMO 2024) are available at https://nucleus.iaea.org/wiser/. Tritium in precipitation data used in this paper are available in Matsumoto et al. (2013); Wang et al. (2016) and Japan Chemical Analysis Center (JCAC) (2024). Monthly tritium in precipitation data from Namie, Fukushima Prefecture, are available upon request to the Yamada et al. (2024) authors. Tritium measurements in Niida River basin are available in Yabusaki et al. (2015) and Ueda et al. (2015). The MIROC5-iso monthly and daily outputs used in this study, the TracerLPM results, and the anthropogenic tritium daily release, based on reconstructed iodine-131 total gas emissions (Katata et al. 2015), are available in the Zenodo database (https://doi.org/10.5281/zenodo.14020821, Cauquoin et al. 2024).

## Code availability

The code for the isotopic version MIROC5-iso, including the tritium branch, is available upon request from the private GitHub repository (https://github.com/yoshimura-laboratory/MIROC5-iso Okazaki and Yoshimura 2019).
