# Supplementary material for: Modeling tritium release to the atmosphere during the Fukushima Daiichi Nuclear Power Plant accident and application to estimating post-accident water system transit times
Source: Environ Sci Pollut Res Int. 2025 Jan 16;32(7):3649–63. doi: 10.1007/s11356-025-35919-1 (PMC11835976; doi:10.1007/s11356-025-35919-1)
Supplement: Supplementary file 1 — (pdf 506 KB) [file 11356_2025_35919_MOESM1_ESM.pdf]

Supplementary Information for "Modeling tritium release to the atmosphere during the Fukushima Daiichi Nuclear Power Plant accident and application to estimating post-accident water system transit times"

Alexandre Cauquoin,*,[1] Maksym Gusyev,[2] Hayoung Bong,[1,3] Atsushi Okazaki,[4] and Kei Yoshimura[1]

[1] *Institute of Industrial Science (IIS), The University of Tokyo, Kashiwa, Japan.*

[2] *Institute of Environmental Radioactivity (IER), Fukushima University, Fukushima, Japan.*

[3] *NASA Goddard Institute for Space Studies, New York, NY, USA.*

[4] *Institute for Advanced Academic Research / Center for Environmental Remote Sensing, Chiba University, Chiba, Japan.*

* E-mail: cauquoin@iis.u-tokyo.ac.jp

Contents of this file

- Figures S1 to S4
- Tables S1 to S4

The Figures S1 and S2 show the model-data comparison for tritium concentration in daily and monthly precipitation using the MIROC5-iso simulations nudged to ERA5. The Figure

S3 is an extension of Figure 6 to the period 1950-2016. The Figure S4 displays the simulated tritium concentrations at the Fukushima groundwater discharge point, as in Figure 6, but by using results from div100 simulation nudged to ERA5. The Tables S1 and S2 show the model-data correlation statistics in tritium concentration in daily and monthly precipitation using the simulations nudged to JRA-55. The Tables S3 and S4 present the same statistics but with the simulations nudged to ERA5.

Additional Supporting Information (Files uploaded separately)

- Caption for Dataset S1: Table of anthropogenic tritium daily release, based on reconstructed iodine-131 total gas emissions from Katata et al. (2015), used as inputs for MIROC5-iso. From left to right, the columns correspond to the date, the minimum and maximum altitudes (m), the total gas iodine-131 emissions in Bq/day and translated in kg of tritium per day, and the calculated anthropogenic tritium releases (kg/day) used as inputs for the simulations div100, div200, div500, and div1000.

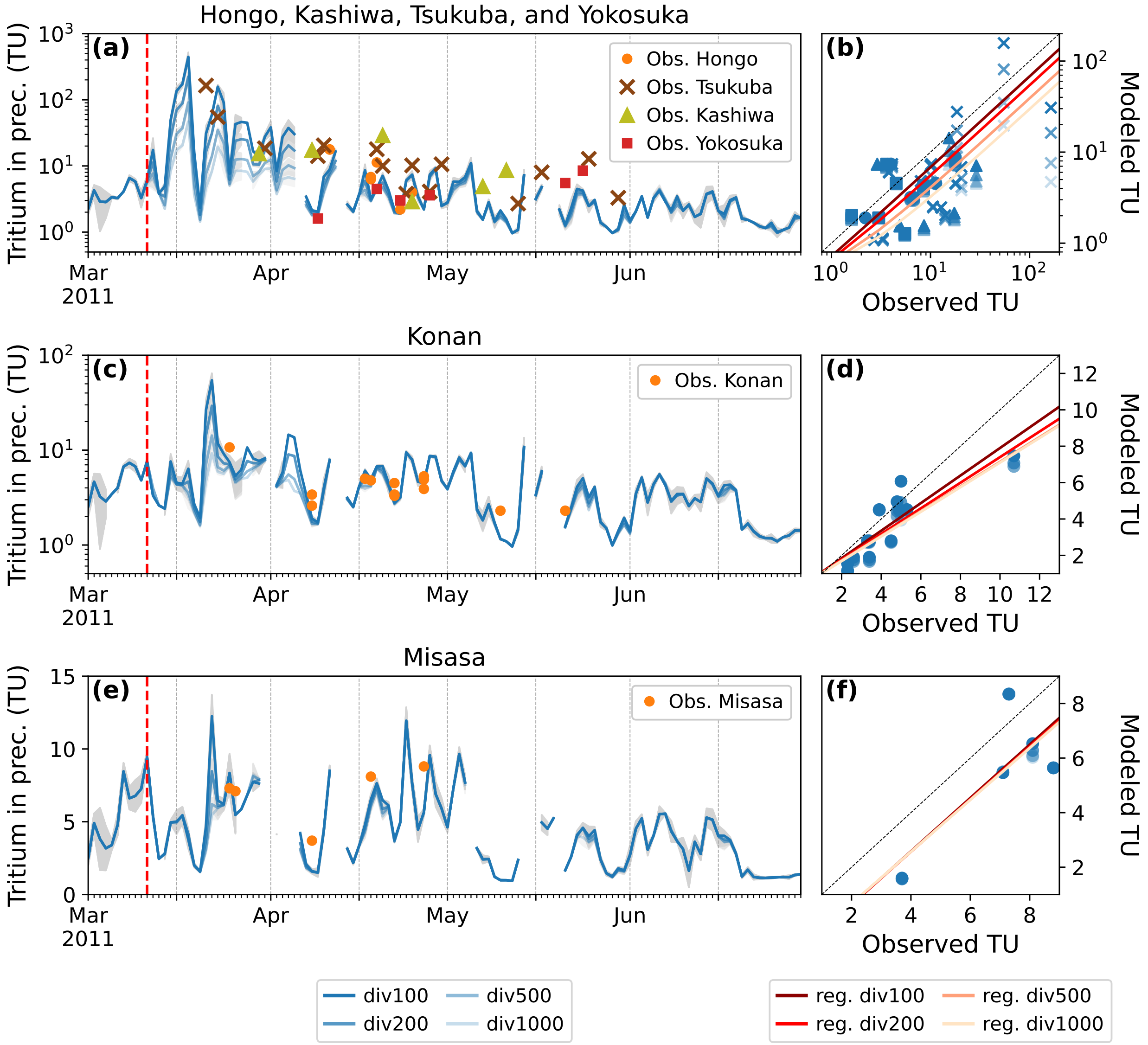

Figure S1: Same as Figure 3 but with MIROC5-iso simulations nudged to ERA5 instead of JRA-55.

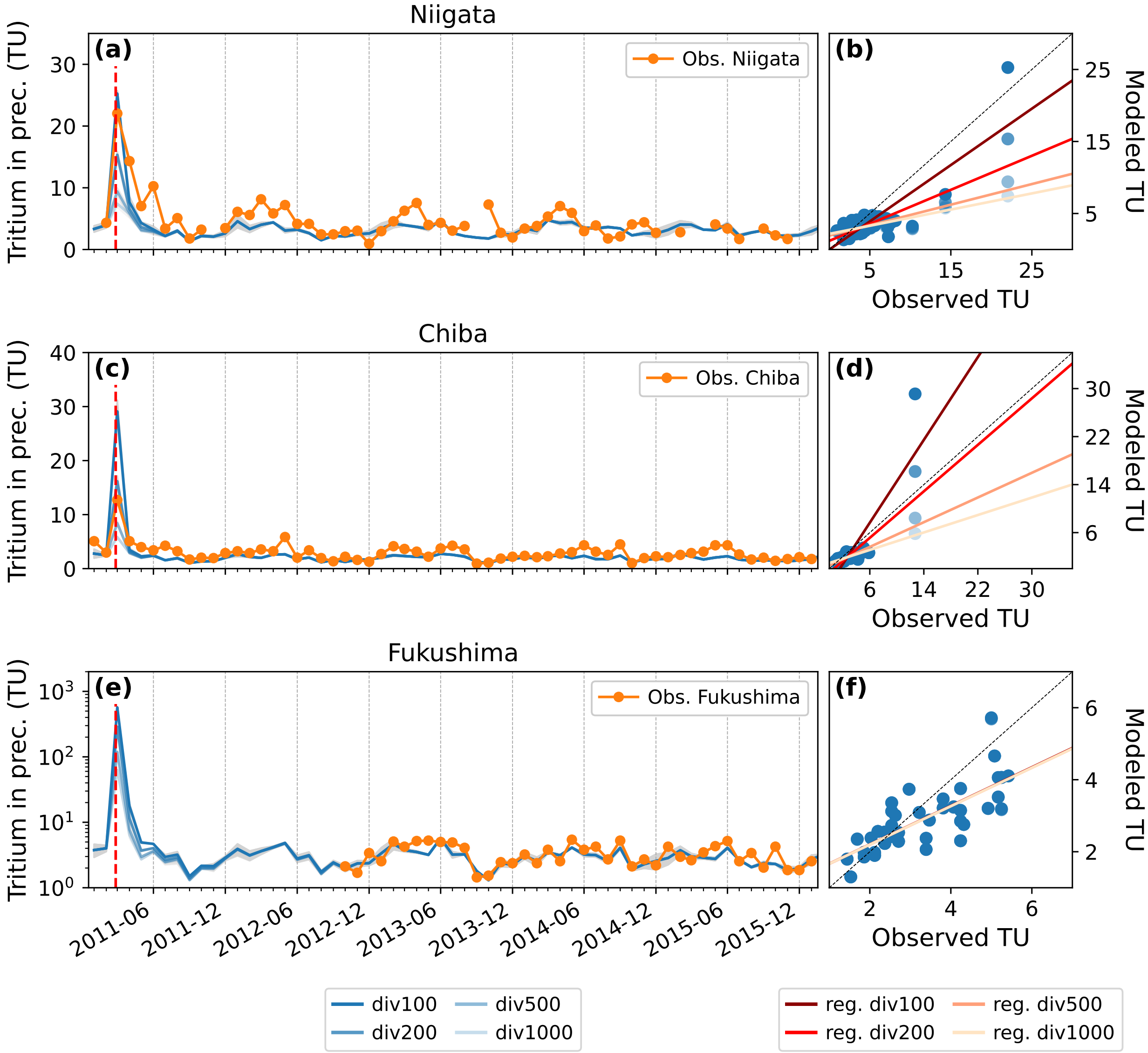

Figure S2: Same as Figure 4 but with MIROC5-iso simulations nudged to ERA5 instead of JRA-55.

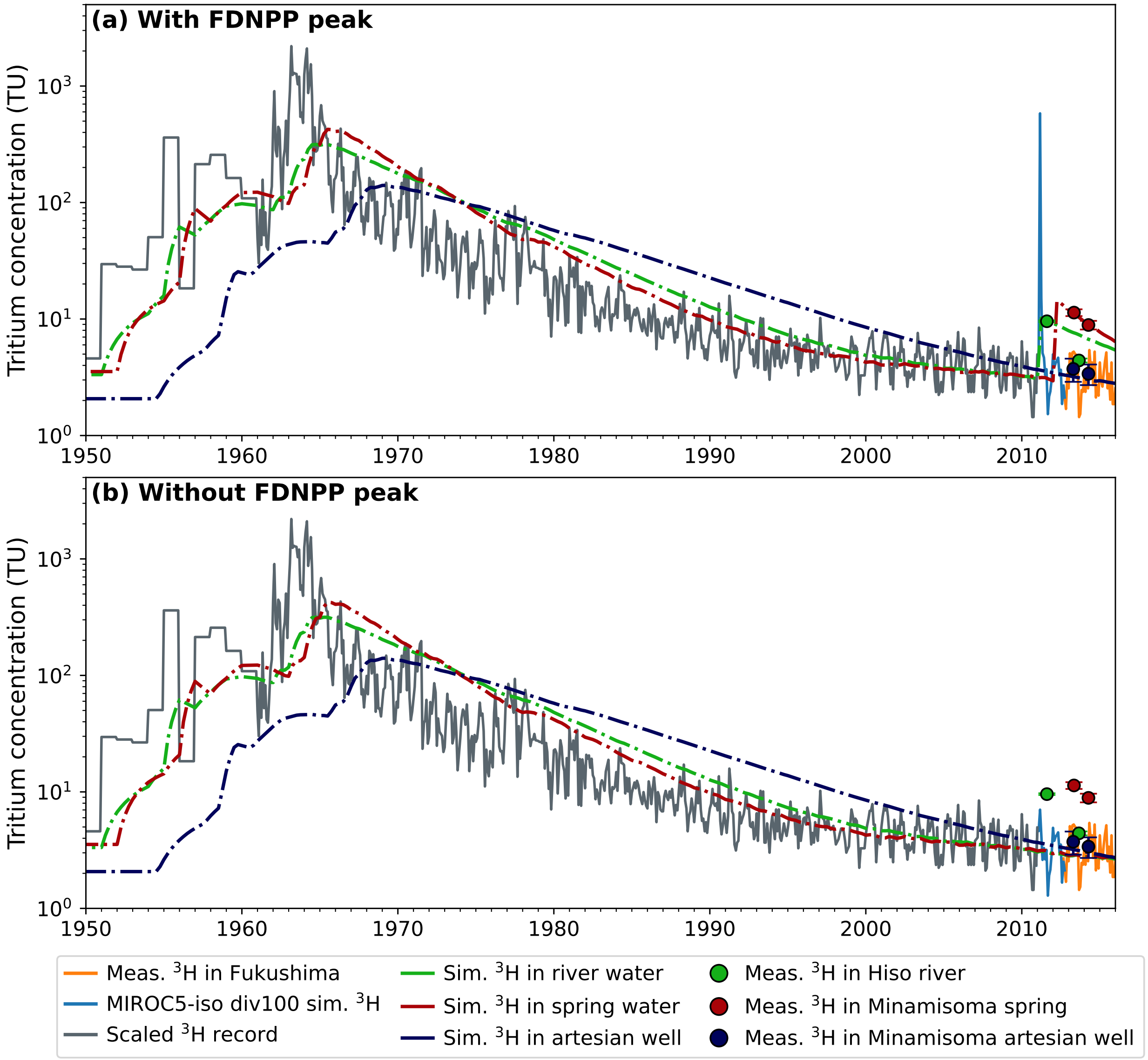

Figure S3: Same as Figure 6 but for the period 1950-2016.

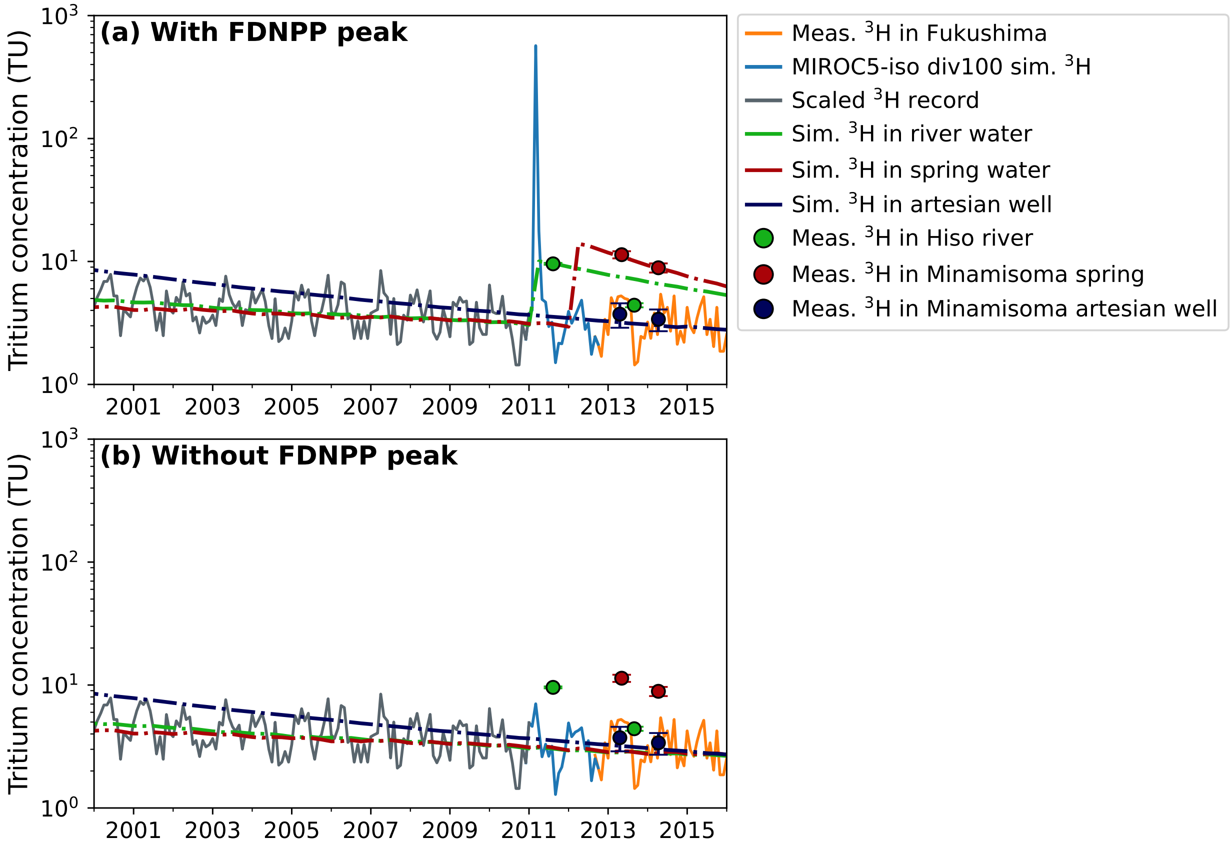

Figure S4: Same as Figure 6 but by using the results from div100 simulation nudged to ERA5 (blue curve) for $C_{in}(t)$.

Table S1: Model-data regression statistics for tritium in daily precipitation shown in Figure 3. Simulations are nudged to JRA-55. The variables a, r, and p are the linear regression gradient, the Pearson correlation coefficient, and the probability value, respectively. Values in red are the best model-data agreements among the simulations. Values in grey are model insignificant model-data correlations ($p > 0.05$).

	div100			div200			div500			div1000		
	a	r	p	a	r	p	a	r	p	a	r	p
**Hongo**	**0.56**	**0.89**	**0.008**	0.42	0.86	0.013	0.34	0.82	0.023	0.31	0.8	0.029
**Kashiwa**	0.22	0.33	0.52	0.12	0.28	0.59	0.05	0.19	0.725	0.03	0.12	0.82
**Tsukuba ($\log_{10}$)**	**0.94**	**0.73**	**0.002**	0.75	0.69	0.004	0.53	0.61	0.015	0.38	0.52	0.047
**Yokosuka**	0.24	0.31	0.554	0.21	0.28	0.586	0.19	0.27	0.608	0.18	0.26	0.623
**Hongo + Kashiwa + Tsukuba + Yokosuka ($\log_{10}$)**	**0.75**	**0.66**	**1.8e-5**	0.61	0.62	9.0e-5	0.44	0.54	9.8e-4	0.33	0.46	6.2e-3
**Konan**	**0.95**	**0.93**	**4.8e-6**	0.77	0.92	7.6e-6	0.66	0.91	2.0e-5	0.63	0.9	3.0e-5
**Misasa**	0.83	0.78	0.118	0.82	0.77	0.131	0.81	0.75	0.146	0.81	0.74	0.149

Table S2: Model-data regression statistics for tritium in monthly precipitation shown in Figure 4. Simulations are nudged to JRA-55. All model-data correlations are significant ($p << 0.05$).

	div100		div200		div500		div1000	
	a	r	a	r	a	r	a	r
**Niigata**	**0.76**	**0.78**	0.45	0.76	0.27	0.67	0.21	0.6
**Chiba**	1.9	0.82	**1.05**	**0.85**	0.55	0.87	0.38	0.82
**Fukushima**	0.51	0.66	0.51	0.66	0.5	0.65	0.5	0.65

Table S3: Model-data regression statistics for tritium in daily precipitation shown in Figure S1. Simulations are nudged to ERA5.

	div100			div200			div500			div1000		
	a	r	p	a	r	p	a	r	p	a	r	p
**Hongo**	**0.35**	**0.76**	**0.048**	0.28	0.69	0.085	0.24	0.64	0.122	0.23	0.62	0.137
**Kashiwa**	0.13	0.26	0.62	0.06	0.16	0.758	0.02	0.07	0.899	0.01	0.03	0.958
**Tsukuba (log_{10})**	**0.87**	**0.71**	**0.003**	0.69	0.67	0.007	0.47	0.57	0.027	0.34	0.46	0.081
**Yokosuka**	0.14	0.14	0.788	0.12	0.13	0.804	0.11	0.12	0.823	0.1	0.11	0.832
**Hongo + Kashiwa + Tsukuba + Yokosuka (log_{10})**	**0.68**	**0.63**	**6.4e-5**	0.54	0.58	3.6e-4	0.39	0.48	3.8e-3	0.29	0.40	1.9e-2
**Konan**	**0.76**	**0.87**	**1.2e-4**	0.7	0.89	4.7e-5	0.68	0.89	3.9e-5	0.68	0.9	3.5e-5
**Misasa**	0.98	0.78	0.121	0.96	0.77	0.125	0.95	0.77	0.131	0.95	0.76	0.133

Table S4: Model-data regression statistics for tritium in monthly precipitation shown in Figure S2. Simulations are nudged to ERA5. All model-data correlations are significant ($p << 0.05$).

	div100		div200		div500		div1000	
	a	r	a	r	a	r	a	r
**Niigata**	**0.78**	**0.83**	0.47	0.83	0.29	0.77	0.22	0.7
**Chiba**	1.72	0.82	**0.97**	**0.86**	0.51	0.9	0.36	0.88
**Fukushima**	0.54	0.76	0.53	0.76	0.53	0.76	0.53	0.76
